# Skeletal muscle mTORC1 regulates neuromuscular junction stability

**DOI:** 10.1002/jcsm.12496

**Published:** 2019-10-25

**Authors:** Martina Baraldo, Alessia Geremia, Marco Pirazzini, Leonardo Nogara, Francesca Solagna, Clara Türk, Hendrik Nolte, Vanina Romanello, Aram Megighian, Simona Boncompagni, Marcus Kruger, Marco Sandri, Bert Blaauw

**Affiliations:** ^1^ Venetian Institute of Molecular Medicine (VIMM) Padova Italy; ^2^ Institute for Genetics Cologne Excellence Cluster on Cellular Stress Responses in Aging‐Associated Diseases (CECAD) Cologne Germany; ^3^ Center for Molecular Medicine (CMMC) University of Cologne Cologne Germany; ^4^ CeSI‐Met—Center for Research on Ageing and Translational Medicine and DNICS, Department of Neuroscience, Imaging and Clinical Sciences University G. d' Annunzio Chieti Italy; ^5^ Department of Biomedical Sciences University of Padova Padova Italy

**Keywords:** mTOR, NMJ, Autophagy, Mitochondrial dysfunction

## Abstract

**Background:**

Skeletal muscle is a plastic tissue that can adapt to different stimuli. It is well established that Mammalian Target of Rapamycin Complex 1 (mTORC1) signalling is a key modulator in mediating increases in skeletal muscle mass and function. However, the role of mTORC1 signalling in adult skeletal muscle homeostasis is still not well defined.

**Methods:**

Inducible, muscle‐specific Raptor and mTOR k.o. mice were generated. Muscles at 1 and 7 months after deletion were analysed to assess muscle histology and muscle force.

**Results:**

We found no change in muscle size or contractile properties 1 month after deletion. Prolonging deletion of Raptor to 7 months, however, leads to a very marked phenotype characterized by weakness, muscle regeneration, mitochondrial dysfunction, and autophagy impairment. Unexpectedly, reduced mTOR signalling in muscle fibres is accompanied by the appearance of markers of fibre denervation, like the increased expression of the neural cell adhesion molecule (NCAM). Both muscle‐specific deletion of mTOR or Raptor, or the use of rapamycin, was sufficient to induce 3–8% of NCAM‐positive fibres (*P* < 0.01), muscle fibrillation, and neuromuscular junction (NMJ) fragmentation in 24% of examined fibres (*P* < 0.001). Mechanistically, reactivation of autophagy with the small peptide Tat‐beclin1 is sufficient to prevent mitochondrial dysfunction and the appearance of NCAM‐positive fibres in Raptor k.o. muscles.

**Conclusions:**

Our study shows that mTOR signalling in skeletal muscle fibres is critical for maintaining proper fibre innervation, preserving the NMJ structure in both the muscle fibre and the motor neuron. In addition, considering the beneficial effects of exercise in most pathologies affecting the NMJ, our findings suggest that part of these beneficial effects of exercise are through the well‐established activation of mTORC1 in skeletal muscle during and after exercise.

## Introduction

In most cell types, the kinase mTOR is a major regulator of cellular metabolism, protein synthesis, and turnover.^1^ It has been shown that mTOR can be found in two different complexes, that is, mTORC1 when bound to the scaffold protein Raptor and mTORC2 when bound to the scaffold protein Rictor. In skeletal muscle, both pharmacological and genetic interventions impinging on mTORC1 signalling have shown that modulation of the activity of this complex can have major effects on increases in muscle size and function. Most of the studies addressing mTORC1 signalling in skeletal muscle have used pharmacological inhibition by treating mice with its specific inhibitor rapamycin. For example, different models of muscle hypertrophy are characterized by an increase in mTOR signalling^2^ and show a significant reduction or even complete ablation of muscle growth when administering rapamycin. We recently showed that this inhibitory effect of rapamycin in skeletal muscle is due to the inhibition of the two best defined mTORC1 targets: S6K1 and 4E‐BP1.^3^ In addition to regulating increases in protein synthesis, multiple reports also link mTORC1 signalling to protein breakdown. Early reports suggested that enhancement of autophagic flux in adult skeletal muscle during catabolic conditions is mainly regulated by FoxO transcription factors,^4^, ^5^ while basal autophagy is modulated by mTORC1.^6^

While these findings clearly show an important role of mTOR signalling during adult muscle hypertrophy, results obtained from transgenic mice show a less straight‐forward role of mTOR signalling in muscle growth. Deletion of Rictor specifically from skeletal muscle does not compromise muscle growth or function, while muscle‐specific ablation of Raptor from birth leads to a myopathy around 8 weeks of age and premature death.^7^ A similar late‐onset myopathy with premature death was observed after deletion of the kinase mTOR from skeletal muscle.^8^ While these results clearly show an important role of mTOR and Raptor in skeletal muscle, it is remarkable that no phenotype was observed during the first 6–8 weeks of postnatal growth, when skeletal muscle undergoes its most pronounced increase in size. This late‐onset myopathy after mTORC1 inhibition, which would suggest an important role for mTORC1 signalling in adult muscle, is in stark contrast to the apparent beneficial effects of administration of rapamycin to aged animals, even prolonging lifespan.^9^

With regard to pathology, reduction of mTORC1 signalling by rapamycin is beneficial for numerous diseases, like certain cancers, aging, or neurological diseases like Huntington's.^10^ However, in some neurodegenerative diseases, like amyotrophic lateral sclerosis (ALS) or spinal and bulbar muscular dystrophy (SBMA), treatment with rapamycin has shown to be detrimental.^11^, ^12^ Interestingly, both of these pathologies show a critical contribution of skeletal muscle to their systemic pathological features. Indeed, activation of mTORC1 signalling by muscle‐specific overexpression of IGF‐1 is sufficient to ameliorate the overall pathogenesis and even prolongs lifespan in both ALS and SBMA.^13^, ^14^ These results suggest that maintaining mTORC1 signalling in skeletal muscle is important for the maintenance of the whole neuromuscular system, at least in certain pathological conditions.

In order to identify the functional role of mTORC1 signalling during adult muscle homeostasis, we generated inducible muscle specific knockout mice in which Raptor or mTOR are acutely and specifically deleted in adult animals. Interestingly, we observed that loss of mTORC1 signalling for 1 month is not sufficient to induce a muscle phenotype. However, adding pharmacological mTORC1 inhibition to Raptor k.o. and mTOR k.o. mice was sufficient to induce a very rapid myopathy, while not affecting wild‐type mice, suggesting the presence of residual mTORC1 signalling in the Raptor k.o. and mTOR k.o. 1 month after deletion, which is sufficient for maintaining muscle homeostasis.

Deletion of Raptor for longer periods, however, leads to a significant myopathy, characterized by central nuclei, mitochondrial dysfunction, and reduced muscle function, but without premature death after 7 months of deletion. Surprisingly, while rapamycin treatment leads to the expected increase in autophagy, long‐term Raptor k.o. mice showed an impairment of both bulk autophagic flux and mitophagy flux. Surprisingly, this block of autophagy and consequently increased mitochondrial dysfunction significantly contributes to an instability of the neuromuscular junction. Indeed, both loss of mTOR and Raptor, or the use of rapamycin, are sufficient to induce the appearance of NCAM‐positive fibres, a marker of fibre denervation, and neuromuscular junction degeneration. Importantly, prolonged loss of mTORC1 leads to muscle fibrillation and a significant NMJ fragmentation. Reactivation of autophagy using the Tat‐beclin1 peptide is able to prevent mitochondrial dysfunction and the appearance of NCAM‐positive fibres. Taken together, these results show that basal mTORC1 signalling in adult muscle fibres is necessary for the maintenance of the NMJ.

## Materials and methods

### Mice generation and treatments

Inducible, muscle‐specific Raptor knockout mice and mTOR knockout mice were generated crossing mice expressing Raptor gene or mTOR gene between two LoxP sites (Raptor^fl/fl^ or mTOR^fl/fl^, Jackson Labs) with mice carrying Cre recombinase fused to a mutated estrogen receptor domain under the control of human skeletal actin promoter.^15^ Tamoxifen‐induced Cre LoxP recombination was activated by oral administration of tamoxifen‐containing chow (Tam400/Cre‐estrogen receptor, Harlan), which was administered for 3 weeks. For 1‐month‐treated Raptor k.o. mice, muscles were collected 1 week after the tamoxifen diet finished, whereas for long‐term Raptor k.o. mice, muscles were collected 7 months from the beginning of the treatment. mTOR k.o. mice were treated for 9 weeks with tamoxifen diet. Adult mice of the same age (3 months old) were used for each individual experiment. Each genotype was compared with Cre‐negative animals of the same genetic background treated with tamoxifen. Rapamycin treatment was performed by intraperitoneal injections at 2 mg/kg body weight every day for 2 weeks. Tat‐beclin1 treatment was performed by intraperitoneal injections of Tat‐beclin1 D11 peptide from Novusbio at 20 mg/kg body weight every day for 2 weeks. Colchicine was used to monitor autophagic flux as described previously.^16^ Briefly, control and Raptor k.o. mice were treated with 0.4 mg/kg of colchicine or vehicle by intraperitoneal injection and starved for 24 h. The treatment was repeated twice, 24 and 12 h prior to muscle collection. Experimental protocols were reviewed and approved by the local Animal Care Committee, University of Padova.

### Immunoblotting and histology

Twenty micrometres cryosections of frozen TA or gastrocnemius muscles were lysed in a buffer containing 50 mM Tris (pH 7.5), 150 mM NaCl, 10 mM MgCl_2_, 0.5 mM DTT, 1 mM EDTA, 10% glycerol, 2% SDS, 1% Triton X‐100, Roche Complete Protease Inhibitor Cocktail, and Roche Phospho‐Stop Phosphatase Inhibitor Cocktail. The samples were immunoblotted and visualized with Clarity Chemiluminescent substrate (Bio‐Rad); 10 μm cryosections of TA or gastrocnemius muscles were stained for H&E, PAS, and for succinate dehydrogenase. Dystrophin was used to determine fibre CSA measurement, by using ImageJ software (National Institutes of Health).

### Antibodies

We used the following antibodies for western blotting: P‐AKT (S473; ref. 4060), P‐S6 (S240/244; ref. 5364), S6 (ref. 2217), AKT (ref. 9272), GSK‐3β (ref. 9315), P‐GSK‐3β (S9; ref. 9322), P‐4E‐BP1 (Ser65; ref. 9451), P‐4E‐BP1 (Thr37/46; ref. 2855), 4E‐BP1 (ref. 9644), RAPTOR (ref. 2280), RICTOR (ref. 2114), mTOR (ref. 2983), P‐AMPK (T172; ref. 2531), P‐ULK1 (Ser757; ref. 6888), SDH (ref. 11998) from Cell Signalling, LC3 (ref. L7543) and p62 (ref. P0067) from Sigma, GAPDH (ref. 8245), Mitoprofile (ref. 110413) and TOM20 (ref. 56783) from Abcam, actin (ref. 56459), and GRP‐75 (ref. 13967) and Porin1 (ref. 390996) from Santa Cruz. For immunofluorescence, LAMP1 (ref. 1D4B), embryonic MyHC (ref. BF‐G6), and types I, IIa, and IIb MyHC (ref. BAD5, SC‐71, and BF‐F3, respectively) were from Developmental Studies Hybridoma Bank, mTOR was from Cell Signalling, distrophin (ref. 15277) from Abcam, NCAM (ref. 5032) was from Millipore, and α‐bungarotoxin Alexa Fluor 555‐conjugate (ref. B35451) was from Invitrogen.

### Quantitative proteomics

Sample preparation and in‐solution digest, frozen tissue was cryogenically ground using a mortar and pestle and resuspended in ice‐cold lysis buffer [modified Ripa: 50 mM Tris/HCl (pH 7.5), 150 mM NaCl, 1% NP‐40, 1 mM EDTA, and 0.1% sodium deoxycholate] with protease inhibitor cocktail. After homogenization on a rotating wheel at 4°C for 30 min and sonication the proteins where precipitated with ice‐cold acetone at −20°C over night. The next day, 50 μg of protein dissolved in 8 M urea were reduced with 5 mM DDT for 60 min, followed by carbamidomethylation with 40 mM iodoacetamide for 45 min in the dark at room temperature. A predigest with the protease LysC (Wako Pure Chemicals Industries) was performed for 3 h followed by a digest with trypsin (Promega) for 16 h. The resulting peptides were separated from salts and detergents using the C18‐based Stop and Go extraction tips.

### LC‐MS/MS analysis

An Easy nLC 1000 ultra‐high performance liquid chromatography coupled to a QExactive Plus Hybrid Quadrupole‐Orbitrap mass spectrometer (Thermo Fisher Scientific) was used for the proteomic analysis with the following settings. Peptides were fractionated using in‐house made 50 cm columns packed with 1.7 μm C18 beads using a binary buffer system, consisting of Buffer A (0.1% FA) and Buffer B (80% ACN in 0.1% FA). All samples were analysed over a 240 min gradient, raising the content of Buffer B from 5% to 34% over 215 min and then from 34% to 55% over 5 min, followed by washing with 90% Buffer B. Spectra for full MS were acquired at a resolution of 70 000 at 200 m/z and the automated gain control target was set to 3 × 10^6^ with a maximum injection time of 20 ms. The dynamic exclusion was set to 20 s. The MS2 measurements were acquired using a resolution of 17 500 at 200 m/z with a top 10 data dependent mode. Here, the automated gain control target was set to 5 × 10^5^ with an injection time of 60 ms. The normalized collision energy in the HCD cell was 25.

#### Data processing

The raw data were analysed using the Andromeda search engine implemented in the MaxQuant software 1.5.3.8.^17^ Parameters in MaxQuant were set to default with trypsin selected as protease for digestion. Match‐between runs and LFQ quantification algorithms were enabled additionally. A mouse database from Uniprot (16.06.17) with contaminants was used for peptide and protein identification. Statistical analysis, GO annotations, *t*‐tests, and data visualization were performed using the Perseus software and the Instant Clue software.^18^

### *In vivo* force measurements

Gastrocnemius muscle force was measured in living mice as previously described.^19^ Briefly, animals were anesthetized and muscle contractile performance was measured *in vivo* using a 305B muscle lever system (Aurora Scientific Inc.). Force was normalized to the muscle mass as an estimate of specific force. Animals were then sacrificed by cervical dislocation, and muscles were dissected, weighted, and frozen.

### Electromyography analysis

Resting EMGraphic activity was recorded by inserting one needle electrode in the middle region of TA muscle and the second electrode close to the tendon region. Ground electrode was placed around the tail. Recordings were made at room temperature (20–22°C). Calibration bars in Raptor k.o. mice is the same as in wild‐type mice.

### Mitochondrial membrane potential analysis and mitoKeima

Mitochondrial membrane potential was measured in isolated fibres from the Flexor Digitorum Brevis (FDB) muscles as previously described.^4^ Briefly, FDB muscles were collected with their tendons and digested in a tube with collagenase (3 mg/mL GIBCO‐Life Technologies) in Dulbecco's modified Eagle's medium (GIBCO‐Life Technologies) for 1 h and 30 min at 37°C. Coverslips were coated with 10% matrigel in Tyrode's buffer. After digestion, the muscles were removed from collagenase, washed to inactivate the collagenase, and dissected. FDB myofibers were placed for 15 min at 37°C in 1 mL Tyrode's buffer and loaded with 2.5 nM tetramethylrhodamine (Molecular Probes), which is a cell‐permeant, cationic, red‐orange fluorescent dye that is readily sequestered by active mitochondria in a potential‐dependent manner. Sequential images of tetramethylrhodamine fluorescence were acquired every 60 s with a 4 × 0.5 UPLANSL N A objective (Olympus). At the times indicated by arrows, oligomycin (Olm, 5 μM, Sigma) or the protonophore carbonyl cyanide‐p‐trifluoromethoxyphenylhydrazone (FCCP, 4 μM) (Sigma) was added to the cell culture medium.

To analyse mitophagy flux, we used mitoKEIMA. Electroporation experiments were performed on FDB muscles from wild‐type and knockout animals. Muscles were analysed 12 days later. FDB muscles were collected in 1% P/S Dulbecco's modified Eagle's medium, dissociated and plated on glass coverslips coated with 10% Matrigel in Tyrode's salt solution (pH 7.4). Mitochondria‐targeted mitoKeima plasmid (mito‐Keima) (MBL International) was used to monitor mitophagy in transfected FDB single fibres. mito‐Keima is a coral‐derived protein that exhibits both pH‐dependent excitation and resistance to lysosomal proteases. These properties allow rapid determinations to whether the protein is in mitochondria or the in lysosome.^20^ In fluorescence microscopy, ionized Keima is detected as a red fluorescent signal at acidic pH (lysosome) and neutral Keima as a green fluorescent signal at higher pH (mitochondria). Fluorescence of mito‐Keima was imaged in two channels via two sequential excitations (458 nm, green, and 561 nm, red) and using a 570‐ to 695‐nm emission range. The level of mitophagy was defined as the total number of red pixels divided by the total number of all pixels.

### Mitochondria isolation

Muscle mitochondria from the indicated genotype were isolated from quadriceps muscles as described.^21^ For western blot analysis, mitochondria were lysed with RIPA buffer, containing 150 mM NaCl, 5 mM EDTA, 50 mM Tris (pH 7.5), 1% NP40, and 0.1% SDS.

### Gene expression analysis

Total RNA was extracted from muscles using TRIzol (Invitrogen). Complementary DNA was generated from 0.4 μg of RNA reverse‐transcribed with SuperScript III Reverse Transcriptase (Invitrogen). cDNA samples were then amplified on the 7900HT Fast Real‐Time PCR System (Applied Biosystems) using the Power SYBR Green RT‐PCR kit (Applied Biosystems). All data were normalized to HPRT and to GAPDH expression.

### Electron microscope analysis

For EM, EDL and Soleus muscles were dissected from sacrificed animals, pinned on a Sylgard dish, fixed at room temperature with 3.5% glutaraldehyde in 0.1 M NaCaCO buffer (pH 7.4), and stored in the fixative at 4°C. Fixed muscles were then post‐fixed in a mixture of 2% OsO_4_ and 0.8% K_3_Fe(CN)_6_ for 1–2 h, rinsed with 0.1 M sodium cacodylate buffer with 75 mM CaCl_2_, en‐block stained with saturated uranyl acetate, and embedded for EM in epoxy resin (Epon 812). Ultrathin sections (~40 nm) were cut in a Leica Ultracut R microtome (Leica Microsystem, Austria) using a Diatome diamond knife (DiatomeLtd. CH‐2501 Biel, Switzerland) and examined at 60 kV after double staining with uranyl acetate and lead citrate, with a FP 505 Morgagni Series 268D electron microscope (FEI Company, Brno, Czech Republic), equipped with Megaview III digital camera (Munster, Germany) and Soft Imaging System (Germany).

### Exhaustion exercise

Raptor knockout and wild‐type mice performed 1 day of concentric exercise on a treadmill (LE 8710 Panlab Technology 2B, Biological Instruments), at increasing velocity, according to the protocol of acute exercise previously described.^22^ Briefly, exercise consists of running at a speed of 17 cm/s for 40 min, 18 cm/s for 10 min, 20 cm/s for 10 min, 22 cm/s for 10 min, and then increasing velocity of 1 cm/s and of 2 cm/s alternatively every 5 min, until exhaustion. Exhaustion was defined as the point at which mice spent more than 5 s on the electric shocker without attempting to resume running. Total running distance and time were recorded for each mouse.

### Statistics

All data are expressed as means ± SEMs. Differences between groups were assessed using Student's *t*‐test and one‐way or two‐way analysis of variance (ANOVA), using Tukey's multiple comparison test. Significance was defined as a *P*‐value of less than 0.05.

## Results

### Acute deletion of Raptor in skeletal muscle for 1 month does not affect muscle size or function

**Figure 1:**
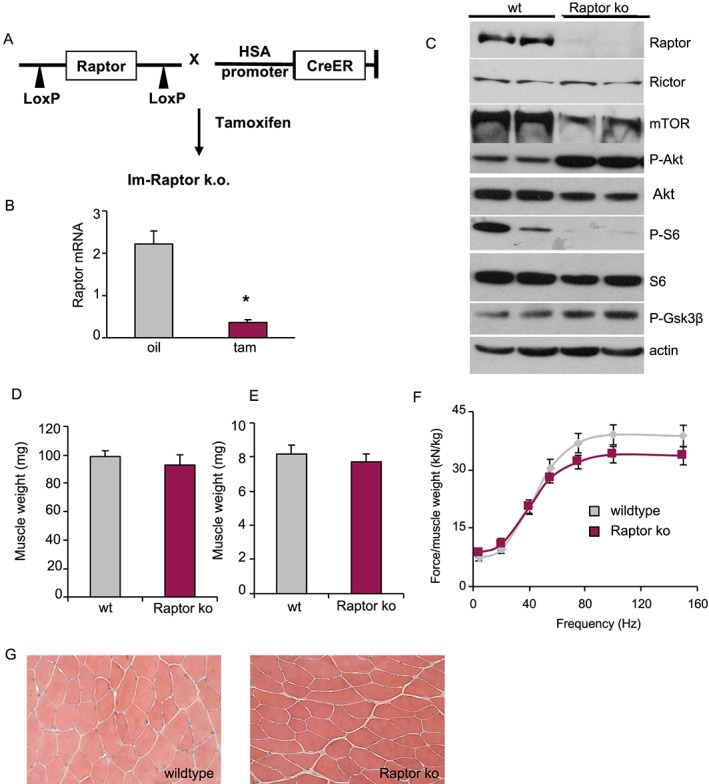
Generation of inducible muscle‐specific Raptor knockout mice (Raptor k.o.). (A) Raptor k.o. mice are generated by crossing two transgenic lines, one expressing two LoxP sites flanking exon 6 of the Raptor gene and one expressing a tamoxifen‐inducible form of Cre (CreER) driven by a human skeletal actin (HSA)‐promoter. Mice were treated with tamoxifen food for 3 weeks to delete the Raptor gene. (B) RT‐PCR for Raptor 4 weeks after the start of tamoxifen treatment shows a strong downregulation of Raptor (*n* = 4 mice per group). (C) Western blot of muscles taken out 4 weeks after the start of tamoxifen treatment. (D, E) Wet weight of gastrocnemius (D) and soleus muscles (E) is not different between groups (*n* = 6 mice per group). (F) Force production of the gastrocnemius muscle does not show any difference between wt and Raptor k.o. muscles (*n* = 4 per group). (G) H&E staining shows no pathological signs 1 month after Raptor deletion. Data are shown as mean ± SEM. Statistical analysis was performed using two‐tailed Student's *t*‐test. Statistical significance: *P < 0.05.

In order to address the role of Raptor, and therefore mTORC1, in adult skeletal muscle, we generated a tamoxifen inducible, muscle‐specific Raptor k.o. mouse. We crossed a line expressing a tamoxifen‐inducible Cre placed under a human skeletal actin promoter, with a line containing two LoxP sites on exon 6 of the Raptor gene (*Figure* 1A). Three‐month‐old mice were placed on tamoxifen food for 3 weeks, and muscles were examined 1 month after the start of treatment. As can be seen in *Figure* 1B and 1C, tamoxifen treatment leads to a very significant reduction in Raptor at both the transcript and protein level. Giving further support for an efficient knockdown of mTORC1, we detected a strong reduction in phosphorylation of RPS6 and 4E‐BP1, and a hyperphosphorylation of Akt. This hyperphosphorylation is in part a consequence of a strong reduction in the destabilizing phosphorylation of S6K1 on IRS‐1,^23^ therefore maintaining total IRS‐1 levels higher in Raptor k.o. muscles (Supporting Information, *Figure* S1A and S1B). Interestingly, loss of Raptor protein corresponds to a strong reduction (around 50%) of total mTOR protein levels, which no longer colocalizes with the lysosomal marker Lamp2 (Supporting Information, *Figure* S1C). One month after tamoxifen treatment, the wet weight of the fast gastrocnemius and slow soleus muscles are unaltered (*Figure* 1D and 1E). Next, we wanted to understand if loss of Raptor affects muscle histology and function. As can be seen in *Figure* 1F and 1G, muscle force *in vivo* produced by the plantar flexors and muscle histology by H&E staining are the same for both groups.

### Rapamycin treatment worsened the phenotype of Raptor or mTOR knockout and caused the onset of myopathic features

**Figure 2:**
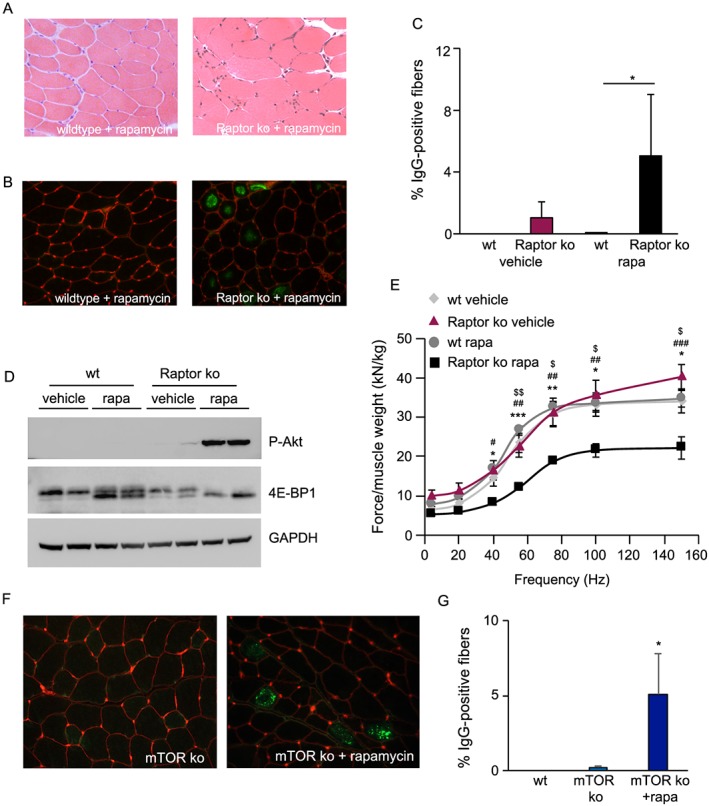
Rapamycin treatment in Raptor k.o. mice induces a very significant myopathy. (A) H&E staining of wild‐type and Raptor k.o. muscles treated for 2 weeks with rapamycin. In Raptor k.o. mice, rapamycin leads to appearance of myopathic features (right panel). (B) Necrotic fibres are identified by a mouse IgG staining, which is a sign of membrane permeability. (C) Quantification of IgG positive myofibers, indicative of fibre necrosis (*n* = 4 muscles/group). (D) Rapamycin treatment leads to a hyperphosphorylation of Akt, with a reduced 4E‐BP1 phosphorylation, underlining the complete mTORC1 inhibition. (E) Normalized force of the gastrocnemius muscle of Raptor k.o. mice is severely reduced after rapamycin treatment, while this does not affect the force in wild‐type mice (*n* = 6–8 per group). (F) Inducible, muscle‐specific knockout of mTOR does not lead to muscle wasting or the appearance of IgG positive fibres. Only after 2 weeks of rapamycin treatment do we observe numerous positive fibres. (G) Quantification of IgG positive myofibers in mTOR k.o. muscles treated with vehicle or rapamycin (*n* = 4 per group). Data are shown as mean ± SEM. Statistical analysis was performed using two‐tailed Student's *t*‐test and two‐way ANOVA when required. Statistical significance: *P < 0.05, **P < 0.01, and ***P < 0.001. With regard to *Figure* 2E, Raptor k.o. rapamycin was compared with *, wild‐type rapamycin; #, Raptor k.o. vehicle; and $, wild‐type vehicle.

The absence of a phenotype in Raptor k.o. muscles 1 month after deletion was surprising, considering the numerous cellular processes regulated, at least in part, by mTORC1. In order to understand if this lack of phenotype was due to some residual mTORC1 signalling in the Raptor k.o. mice, we treated wild‐type and Raptor k.o. mice, 1 month after starting tamoxifen treatment, for 2 weeks with the mTORC1 inhibitor rapamycin. As can be seen in *Figure* 2A, rapamycin treatment leads to a rapid appearance of myopathic features, like central nuclei, in Raptor k.o. muscles, while nothing is seen in wild‐type muscles treated with rapamycin. Also, a staining for mouse immunoglobulins (IgG), which is a good indication of membrane permeability and fibre necrosis, shows a significant increase in Raptor k.o. muscles after rapamycin treatment (*Figure* 2B and 2C). Furthermore, signalling analysis in Raptor k.o. mice after rapamycin treatment shows a very strong increase in Akt phosphorylation, with an even further decrease in phosphorylation of 4E‐BP1, suggesting a more pronounced inhibition of mTORC1 signalling and subsequent reduced negative feedback loop on IRS‐1 (*Figure* 2D). Surprisingly, 2 weeks of rapamycin treatment is sufficient to reduce normalized tetanic muscle force *in vivo* by 45%, while not affecting muscle function in wild‐type mice (*Figure* 2E).

This drastic effect of rapamycin in Raptor k.o. mice can have multiple interpretations: (i) rapamycin has off target effects, which are accentuated in Raptor k.o. mice; (ii) there are Raptor‐independent, mTOR‐dependent effects of rapamycin; and (iii) there is a residual amount of mTORC1 activity, which is sufficient to maintain most of its basic tasks. Because rapamycin is very specific in its effects on mTOR, we wondered if there could be a Raptor‐independent, mTOR‐dependent effect. To address this issue, we generated mice in which we crossed the inducible, muscle‐specific Cre‐line to a line expressing two LoxP sites flanking exon 1‐5 of the mTOR gene. As can be seen in Supporting Information, *Figure* S2A and S2B, 2 months after tamoxifen treatment, these mice showed a significant reduction in mTOR mRNA and protein levels. Interestingly, also the deletion of mTOR did not lead to a major muscle phenotype as muscle weight (Supporting Information, *Figure* S2C) was not affected. However, treating these mice for 2 weeks with rapamycin leads to a very rapid appearance of IgG‐positive fibres (*Figure* 2F and 2G). The lack of any obvious phenotype after inducible deletion of either Raptor or mTOR from adult skeletal muscle and the very rapid appearance of a myopathic phenotype after rapamycin treatment in both strains are suggestive of the presence of a residual amount of mTORC1, which can maintain muscle mass and force in homeostatic conditions.

### Long‐term deletion of Raptor leads to fibre regeneration, mitochondrial dysfunction, and reduced force production

**Figure 3:**
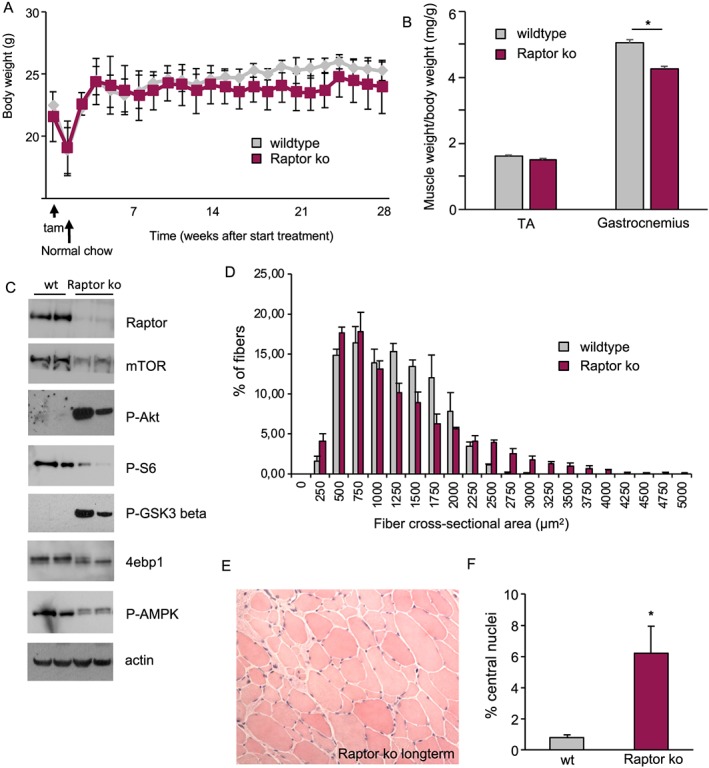
Long‐term deletion of Raptor does not reduce body weight or longevity. (A) Three‐month‐old mice were placed on tamoxifen food for 3 weeks after which they were placed on regular chow. Mice were injected every month with an injection of tamoxifen for three consecutive days to avoid re‐introduction of Raptor via satellite cell proliferation (*n* = 6 per group). (B) Average muscle ratio of weight/body weight of TA and gastrocnemius muscles 6 months after Raptor deletion (*n* = 6 per group). (C) Analysis of Akt‐mTOR signalling pathway shows increased phosphorylation of Akt and GSK‐3β in long‐term Raptor k.o. muscles. (D, E, F) Histological analysis 6 months after Raptor deletion shows fibre size heterogeneity and central nuclei (*n* = 6 per group). Data are shown as mean ± SEM. Statistical analysis was performed using two‐tailed Student's *t*‐test. Statistical significance: *P < 0.05.

It has been reported that loss of Raptor from skeletal muscle leads to a myopathic phenotype, which starts manifesting itself after the postnatal growth period, culminating in premature death between 5–7 months of age.^7^ In order to understand if also inducible deletion of Raptor from adult skeletal muscle leads to a myopathy at later timepoints, we deleted Raptor from 3‐month‐old mice and analysed muscles after 7 months. Interestingly, loss of Raptor did not lead to a decrease in body weight and only minor reductions in muscle weight after 7 months (*Figure* 3A and 3B). The signalling changes observed after 1 month of Raptor deletion (*Figure* 1C) were more pronounced after 7 months, showing a hyperactivation of Akt and a marked increase in GSK‐3β phosphorylation (*Figure* 3C). Furthermore, when analysing muscle histology by H&E staining, we observed a heterogeneous fibre size distribution with a significant increase in smaller and larger fibres in Raptor k.o. muscles (*Figure* 3D and 3E). Also, 6.1 ± 1.2% of fibres in the tibialis anterior muscle showed the presence of centrally nucleated fibres (*Figure* 3F).

**Figure 4:**
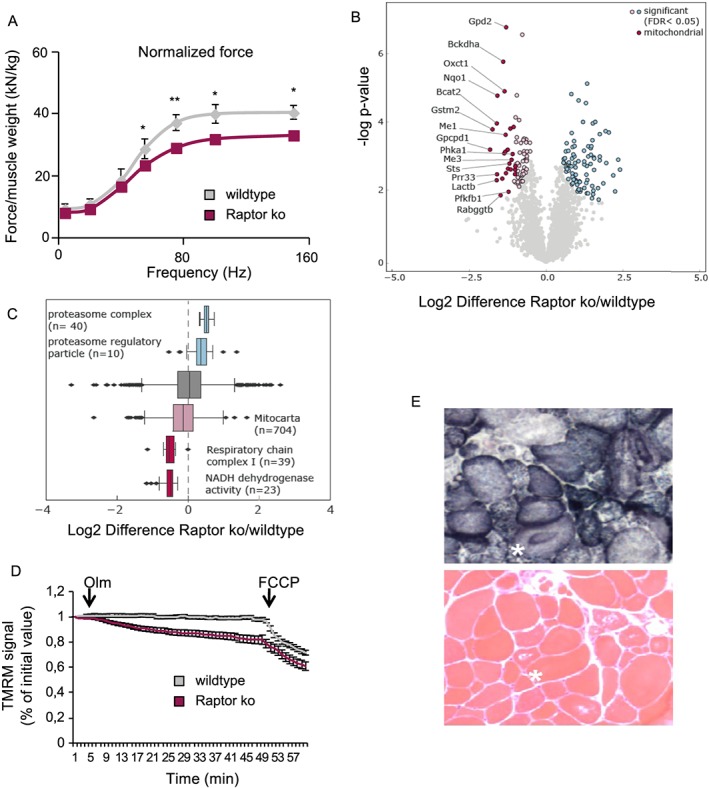
Long‐term deletion of Raptor leads to mitochondrial dysfunction with reduced force production. (A) Normalized force measured *in vivo* is significantly reduced in Raptor k.o. mice (*n* = 6 per group). (B) Volcano plot of differentially regulated proteins in Raptor k.o. muscles 7 months after the deletion of Raptor (*n* = 3). Significantly regulated proteins are marked in blue and light red (FDR < 0.05, s0 = 0.1, number of permutations: 500). Mitochondrial proteins are highlighted in dark red. (C) Box plot of log2 Raptor k.o./wild‐type ratios of proteins associated with the proteasome, the respiratory chain complexes or the mitochondrion. (D) Mitochondrial dysfunction revealed by TMRM. Oligomycin (Olm) and the protonophore FCCP were added at the indicated timepoints (*n* = 30/group). (E) Mitochondrial morphology is drastically altered in Raptor k.o. muscles. Data are shown as mean ± SEM. Statistical analysis was performed using two‐tailed Student's *t*‐test. Statistical significance: *P < 0.05 and **P < 0.01.

The previously reported conditional muscle‐specific Raptor k.o. mice showed a lethal myopathy, despite the fact that grip strength and specific muscle force of either EDL or soleus muscles was unaffected.^7^ To assess if inducible deletion can unmask a role for Raptor in muscle force production, we analysed the force production in the gastrocnemius muscle using an i*n vivo* approach. As can be seen in *Figure* 4A, normalized tetanic force is significantly reduced in Raptor k.o. mice. To better understand why force is depressed in Raptor k.o. muscles, we performed a quantitative proteomics analyses at 1 and 7 months after deletion of Raptor. In line with the lack of phenotype at 1 month after Raptor deletion, we did not find any significant alterations in the proteome at this timepoint (Supporting Information, *Figure* S4A). However, 7 months after deletion, we found 162 proteins which were significantly dysregulated in Raptor k.o. muscles (*Figure* 4B). When performing a GO‐term enrichment on this group of proteins, many of the downregulated proteins were linked to mitochondrial function (*Figure* 4C). This important downregulation of mitochondrial proteins was somewhat unexpected, because we did not observe major changes in total mitochondria, as assessed by western blotting for mitoprofile, SDH and TOM20 (Supporting Information, *Figure* S4B). While mitochondrial content does not seem to be altered in Raptor k.o. muscles, we wondered if mitochondrial function might be affected. Indeed, when measuring mitochondrial membrane potential from isolated single fibres (*Figure* 4D), we found a significant reduction in mitochondrial function in Raptor k.o. mice. Also, histological analyses of Raptor k.o. muscles showed a highly anomalous distribution when performing a SDH staining (*Figure* 4E). The reduced mitochondrial function is also revealed by a reduced capacity to perform on the treadmill, as Raptor k.o. mice run 41 ± 3% less than their wild‐type littermates (Supporting Information, *Figure* S4C).

### Long‐term Raptor deletion leads to a block in autophagic flux

**Figure 5:**
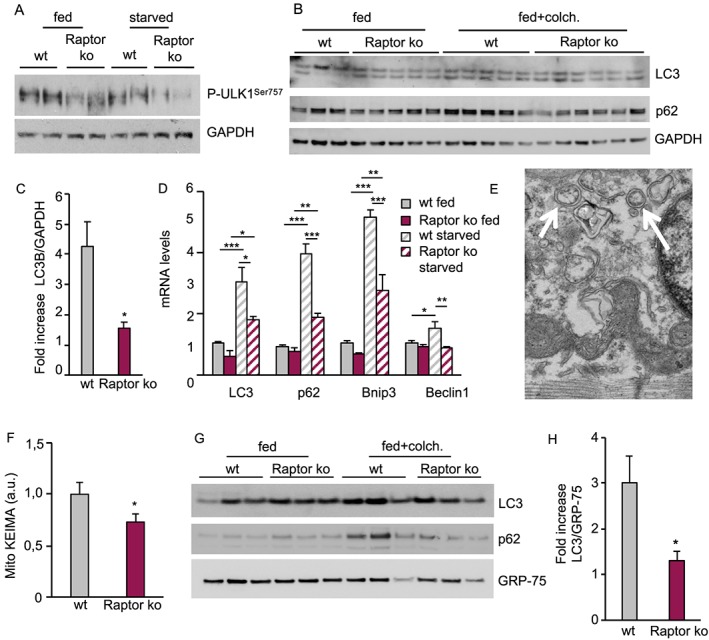
Long‐term deletion of Raptor leads to a block in autophagic flux. (A) Reduction in ULK1 phosphorylation on its inhibitory mTOR site. (B) Western blotting for LC3, p62, and GAPDH after treatment with the autophagy inhibitor colchicine shows a reduction in autophagic flux as evidenced by the lack of increased LC3 lipidation after 24 h of colchicine treatment. (C) Relative increase in LC3 lipidation after colchicine treatment (*n* = 4–5 muscles/group). (D) Strong reduction in the transcription of autophagy‐related genes under basal and starvation conditions at 1 month after deletion (*n* = 6–7 per group). (E) Electron microscopy images showing the accumulation of numerous intracellular vesicles (white arrows). (F) MitoKEIMA measurement shows a reduced mitophagy after long‐term Raptor deletion (*n* = 4–5 muscles/group). (G) Western blot for LC3 and p62 on the isolated mitochondrial fraction shows an impaired mitophagic flux in Raptor k.o. muscles. GRP‐75 was used for normalization of mitochondrial content (*n* = 4 muscles/group). (H) Quantification of LC3 in isolated mitochondria from wt and Raptor k.o. mice (*n* = 4 muscles/group). Data are shown as mean ± SEM. Statistical analysis was performed using two‐tailed Student's *t*‐test and two‐way ANOVA when required. Statistical significance: *P < 0.05, **P < 0.01, and ***P < 0.001.

Mitochondrial dysfunction can occur when organelle turnover is reduced. In addition to regulating protein synthesis, mTORC1 activation is also a well‐established inhibitor of autophagy, which is critical for mitochondrial turnover. In order to assess how long‐term loss of Raptor affects muscle autophagy, we evaluated the inhibitory phosphorylation of mTORC1 on ULK1, an important step in autophagy induction. As can be seen in *Figure* 5A, loss of Raptor leads to a significant reduction in ULK1 phosphorylation in both fed and starved conditions, suggesting an induction of the autophagic program. However, when analysing autophagic flux by using the inhibitor colchicine, we did not observe an increase of p62 and lipidated LC3 in Raptor k.o. (*Figure* 5B). Indeed, quantification of the fold increase of LC3‐II in Raptor k.o. muscles after colchicine treatment is significantly reduced compared with wild‐type muscles (*Figure* 5C). Interestingly, transcriptional regulation of autophagy‐related genes, like LC3, p62, Bnip3, and Beclin1, were markedly reduced in Raptor k.o. mice in both fed and starved conditions (*Figure* 5D and Supporting Information, *Figure* S5). Also, analyses by electron microscopy showed clear accumulation of vesicles and altered mitochondria in Raptor k.o. muscles (*Figure* 5E). Next, we analysed if this block in bulk autophagy also affected mitochondrial turnover. We transfected fibres with the mitophagy probe mitoKEIMA and observed a significant reduction in mitophagic flux in Raptor k.o. muscles (*Figure* 5F). These results were confirmed by performing western blots for LC3 and p62 on isolated mitochondria, showing no increase in p62 and LC3 on mitochondria of Raptor k.o. muscles after colchicine treatment (*Figure* 5G and 5H).

### Loss of muscle mTORC1 leads to fibre denervation

**Figure 6:**
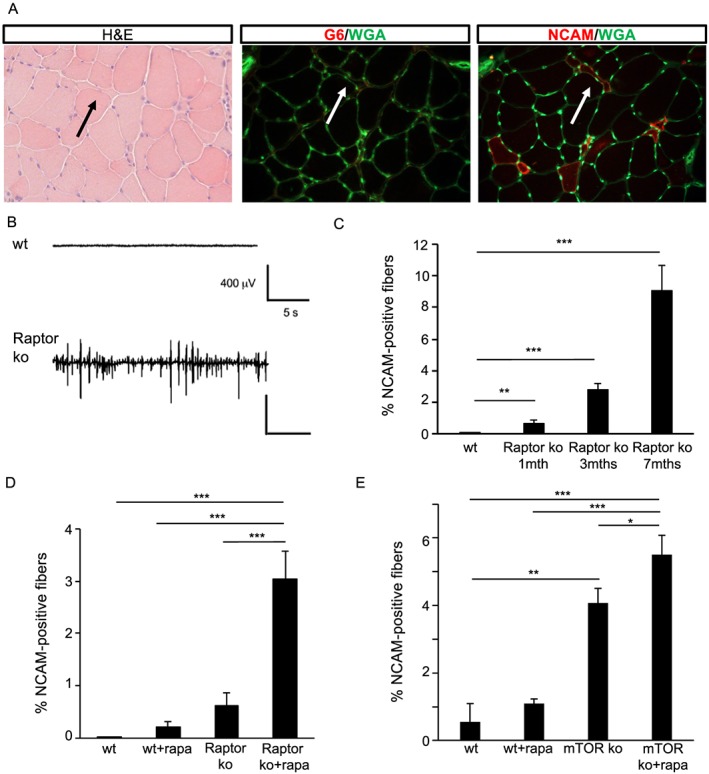
Loss of mTORC1 signalling leads to the appearance of denervated muscle fibres. (A) H&E staining shows numerous small, angulated fibres, which are positive for a marker of denervated fibres (NCAM) and negative for embryonic myosin heavy chain (G6), a marker of regenerating fibres. (B) Long‐term Raptor k.o. muscles show spontaneous fibrillations, which are completely absent in wt mice. Calibration bars in Raptor k.o. mice is the same as in wild‐type mice (*n* = 4 muscles/group). (C) The percentage of NCAM‐positive fibres increases over time in the Raptor k.o. mice. (D) Number of NCAM‐positive fibres increases in Raptor k.o. mice after 2 weeks of rapamycin treatment (*n* = 5–7 muscles/group). (E) Also, mTOR k.o. mice show a significant increase in NCAM‐positive fibres, which is further increased by rapamycin treatment. Data are shown as mean ± SEM. Statistical analysis was performed using two‐tailed Student's *t*‐test and two‐way ANOVA when required. Statistical significance: *P < 0.05, **P < 0.01, and ***P < 0.001.

One of the most striking features of long‐term Raptor deletion is the presence of numerous small, angulated fibres (*Figure* 6A). Interestingly, in most cases, these small fibres did not show centrally localized nuclei, suggesting they were undergoing atrophy and not regeneration. To confirm that these fibres were undergoing atrophy and not regeneration, we stained sections for a marker of muscle regeneration (embryonic myosin, G6) and for a marker of fibre denervation (NCAM). As can be seen in *Figure* 6A, the small, angulated fibres in long‐term Raptor k.o. muscles were positive for NCAM and not for embryonic myosin. Importantly, when we examined the whole section of the tibialis anterior muscle, we found numerous fibres which were positive for NCAM (Supporting Information, *Figure* S6). While NCAM is a good marker for denervated fibres, it is well known that functional denervation induces an increase in spontaneous electrical activity of muscle fibres. Indeed, when we measured the resting EMG activity by placing a recording electrode in the mid‐belly of the TA, we found significant fibrillation in Raptor k.o. mice, which is completely absent in wild‐type animals (*Figure* 6B). Next, we wanted to see if fibre denervation is progressive and correlated with mTORC1 activity levels. To monitor this, we took out muscles at different timepoints after Raptor deletion. As can be seen in *Figure* 6C, genetic reduction of mTORC1 by Raptor deletion leads to a progressive increase in denervated fibres. To understand if also an abrupt, complete ablation of muscle mTORC1 can lead to fibre denervation, we deleted Raptor from muscles for 1 month and then treated mice for 2 weeks with rapamycin. As can be seen in *Figure* 6D, the number of NCAM positive fibres increases significantly as compared with vehicle treated Raptor k.o. muscles. Interestingly, rapamycin treatment in wild‐type animals also leads to the appearance of NCAM‐positive fibres. In order to understand if this effect is due to reduced mTOR signalling and not to off target effects, we repeated these experiments in inducible mTOR k.o. mice. In these mice, we found an even more pronounced increase in NCAM positive fibres after rapamycin treatment (*Figure* 6E).

**Figure 7:**
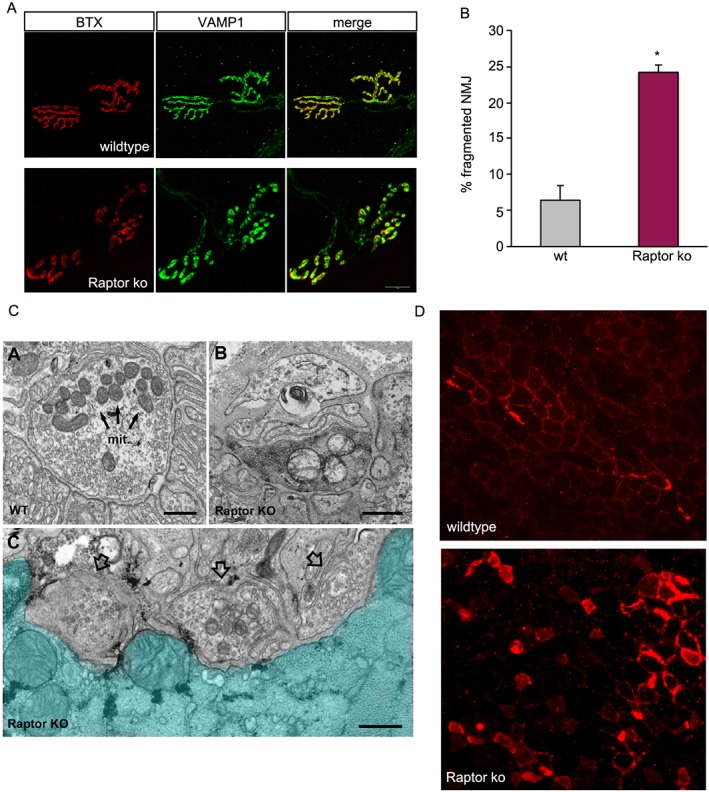
Reduced mTORC1 signalling leads to neuromuscular junction fragmentation. (A) NMJs in wt muscles show their normal pretzel‐like shape. Raptor k.o. mice show numerous NMJs in which both presynaptic (VAMP1) and postsynaptic [acetylcholine receptor (BTX)] structures accumulate in individual, non‐connected aggregates. (B) Quantification of the percentage of fragmented NMJs in wt and long‐term Raptor k.o. mice (*n* = 4 mice/group). (C) Electron microscope images of NMJs in wild‐type and Raptor k.o. muscles. Note the altered presynaptic structures in Raptor k.o. muscles. (D) Staining for acetylcholinesterase in wild‐type and Raptor k.o. muscles. Data are shown as mean ± SEM. Statistical analysis was performed using two‐tailed Student's *t*‐test. Statistical significance: *P < 0.05.

Next, we analysed the histology of the neuromuscular junction in the most affected group, that is, after 7 months of Raptor deletion. In the images shown in *Figure* 7A, the pretzel‐like shape of the NMJ in wild‐type muscles in both the presynaptic (VAMP1 in green) and postsynaptic (AChR in red) side can be clearly seen. In mice lacking Raptor, the pretzel‐like shape is strongly compromised, as multiple NMJs show a discontinuous staining for AChR, with a more cluster‐like distribution. Quantification of the number of fragmented NMJs showed a significant increase in Raptor k.o. muscles (*Figure* 7B). Interestingly, images taken by electron microscopy show not only drastic modifications in postsynaptic clefts but also major alterations in the motor neurons, suggestive of retrograde effects of loss of muscle mTORC1 signalling (*Figure* 7C). In addition to finding markers like NCAM, we also see a significant number of fibres positive for acetylcholinesterase (AchE) in Raptor k.o. muscles (*Figure* 7D), something also observed in a rare human disease called X‐linked myopathy with excessive autophagy, which is characterized by an increased activation of autophagy, but with a late block in autophagic flux.^24^

**Figure 8:**
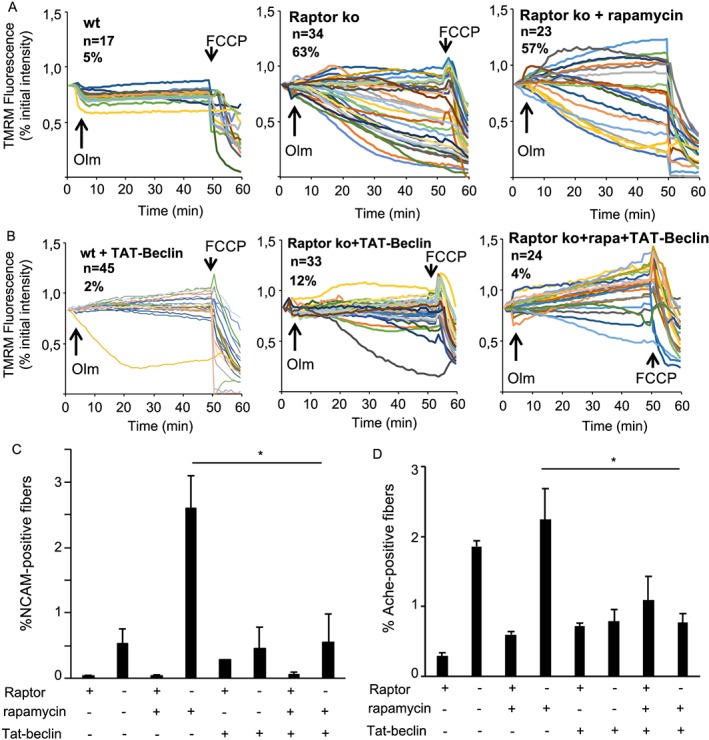
Reactivation of autophagy by Tat‐beclin1 prevents the appearance of NCAM‐positive fibres in Raptor k.o. muscles. (A) TMRM measurement of mitochondrial membrane potential in isolated fibres from the FDB muscle. Raptor k.o. fibres, both with and without rapamycin treatment, show a significant mitochondrial depolarization after oligomycin addition. (B) Mice treated for 2 weeks daily with Tat‐beclin1 completely prevented the mitochondrial dysfunction in Raptor k.o. fibres. (C, D) Gastrocnemius muscles taken from mice treated for 2 weeks with rapamycin and Tat‐beclin1 and stained for NCAM or AchE. No increase in NCAM or AchE staining in Raptor k.o. mice after co‐treatment with rapamycin and Tat‐beclin1 (*n* = 4–6 muscles/group). Data are shown as mean ± SEM. Statistical analysis was performed using two‐tailed Student's *t*‐test and two‐way ANOVA when required. Statistical significance: *P < 0.05.

Because mitochondrial dysfunction and a block in autophagy are linked to NMJ dysfunction,^25^, ^26^ we examined if reactivating autophagy in Raptor k.o. muscles could maintain NMJ stability. To do this, we performed an acute inhibition of mTORC1 signalling by treating Raptor k.o. mice for 2 weeks with rapamycin, while simultaneously administering the autophagy activating peptide Tat‐beclin1.^27^ As can be seen in *Figure* 8A, isolated fibres taken from Raptor k.o. mice show a very significant increase in mitochondrial depolarization after oligomycin addition, either in the presence or absence of rapamycin. Surprisingly, co‐treatment with Tat‐beclin1 is sufficient to completely prevent this mitochondrial dysfunction in both groups (*Figure* 8B). More importantly, when quantifying the number of NCAM‐positive and AchE‐positive fibres in the various groups, we find that Tat‐beclin1 is able to completely prevent the increase in NCAM‐positive and AchE‐positive fibres in Raptor k.o. muscles after rapamycin treatment (*Figure* 8C and 8D). Taken together, these results suggest that loss of mTORC1 leads to a block in autophagy and mitochondrial dysfunction, which causes NMJ instability and the appearance of markers of denervation.

## Discussion

One of the major players in the regulation of adult muscle mass and function is mTORC1.^28^ Despite its critical role in major processes, like protein synthesis, autophagy, and many aspects of muscle metabolism, the role of mTORC1 in adult muscle homeostasis has not been elucidated. Here, we generated muscle‐specific, inducible Raptor and mTOR k.o. mice to address this issue. We find that mTORC1 plays a major role in the maintenance of the neuromuscular junction, in part through its critical role in preventing mitochondrial dysfunction and maintaining basal muscle autophagy. This link between muscle mTORC1 and fibre innervation is of great interest, as exercise is able to strongly activate mTORC1 signalling in muscle fibres. Considering the beneficial effects of exercise on numerous diseases which show a compromised NMJ, like aging or cancer, it is suggestive to think that part of the beneficial effects of exercise on the NMJ are caused by maintaining mTORC1 activation levels in skeletal muscle.

### Reduced mTOR signalling is able to maintain muscle homeostasis in the short term

A surprising finding in this study was the fact that deletion of either Raptor or mTOR from skeletal muscle for 1 month does not lead to a muscle myopathy or muscle wasting. It was reported that electroporation of a siRNA leads to a reduction in fibre size 4 to 6 weeks after electroporation.^29^ The discrepancy in the two observations can most likely be explained by the different approach to reduce Raptor levels. While in our mouse model, we induce deletion without creating muscle damage, electroporation is known to cause significant muscle damage. Considering our finding that reduced mTORC1 signalling by knockdown of Raptor can increase the susceptibility of muscle fibres to additional stresses that affect mTORC1 signalling (like inflammation), it is likely that the difference in approach contributed to the apparent discrepancies in fibre size. Indeed, the lack of atrophy seen in our Raptor k.o. mouse was also reported by another group which generated an inducible, muscle‐specific Raptor k.o. mouse.^30^

While we did not observe any basal atrophy, we do observe an important number of regenerating fibres and a significant drop in muscle force when administering rapamycin for only 2 weeks in Raptor k.o. mice. While these observations can have multiple interpretations, the most likely explanation is that low levels of residual mTORC1 can maintain a lot of the basic cell functions. Indeed, the muscle‐specific deletion of mTOR or Raptor from birth does not impair postnatal growth. Considering that this early growth period is characterized by an important activation and fusion of satellite cells, it is possible that these newly added myonuclei, in which mTOR and Raptor are not deleted, maintain mTORC1 levels sufficiently high to sustain muscle growth. Indeed, the combination of muscle‐specific deletion of mTOR, with the concomitant expression of a kinase inactive form of mTOR, is sufficient to significantly reduce body growth and lead to premature death around 8 weeks of age.^31^ This suggests a dominant‐negative effect of kinase‐inactive mTOR, reducing residual mTOR signalling, similar to what we observed in our Raptor k.o. mice after rapamycin treatment. The reason for the residual amount of Raptor 1 month after the start of tamoxifen treatment could be due to the fact that the Cre requires more time to reduce Raptor levels to a level that is sufficient to reveal a marked phenotype.^32^ Considering the fact that muscle fibres have hundreds of individual nuclei, it is a challenging tissue to generate an efficient knockdown in all nuclei in relatively short‐time frames. In support of this interpretation, deletion of Raptor for longer time periods leads to a more pronounced reduction of mTORC1 signalling. Indeed, activation of the negative feedback loop of S6K1 on IRS‐1 is significantly increased compared with deletion for 1 month, as evidenced by the hyperactivation of Akt and GSK‐3β. The deleterious effect of rapamycin at 1 month after mTORC1 reduction suggests that rapamycin blocks the residual mTORC1 present, leading to the drastic muscle pathology observed in these mice. We cannot exclude a possible off‐target effect of rapamycin, as rapamycin has also been linked to an inhibitory effect on RyR by sequestering FKBP12.^33^ However, the fact that the deleterious effects of rapamycin are observed in both mTOR k.o. and Raptor k.o. are suggestive that this only occurs when basal mTORC1 signalling is already significantly reduced. Furthermore, the increased pathological features observed in long‐term Raptor k.o. muscles, like glycogen accumulation, force reduction, and fibre denervation, are similar to those observed when treating these two transgenic mouse models at early timepoints for 2 weeks with rapamycin. A similar progressive decline in muscle function was seen when ablating Raptor from cardiac myocytes. Initially, no apparent histological and functional phenotype was observed, despite a complete reduction of mTORC1 signalling. However, deletion for up to 6 weeks leads to a reduction in ejection fraction accompanied by premature mortality.^34^

Taken together, our results suggest that reduced mTORC1 activation levels are able to maintain skeletal muscle homeostasis for short periods; however, complete inhibition leads to a very rapid loss in muscle histology and function.

### Long term mTORC1 inhibition leads to a marked myopathy with impaired autophagic flux

One interesting finding from conditional muscle‐specific Raptor and mTOR k.o. mice is that the loss of muscle mTORC1 signalling reduces longevity. Indeed, no conditional Raptor k.o. mice survive for more than 5–6 months. This reduced survival, however, is difficult to explain by muscle dysfunction, as normalized force and resistance to fatigue are not reduced, and muscle atrophy in the diaphragm is less pronounced than in other myopathies, which are compatible with life.^7^, ^8^ Surprisingly, we do not observe any premature death for up to 7 months after Raptor deletion, despite the presence of muscle atrophy and a reduced specific force. These results suggest that a normal activation of muscle mTORC1 signalling in the first 3 months of life can prevent, presumably, systemic alterations which affect longevity after mTORC1 deletion at embryonic stages. Indeed, conditional Raptor k.o. mice show a reduction in cardiac weight and epidydimal fat mass, something not observed in our inducible Raptor k.o. mice 7 months after deletion^7^ (Supporting Information, *Figure* S3).

The role of mTORC1 signalling in the regulation of autophagy in skeletal muscle has been debated. More than 10 years ago, it was shown that FoxO transcription factors are able to induce autophagy, independently of mTORC1 signalling.^4^ A more recent study, however, showed that autophagy can be blocked by increased mTORC1 signalling, even in the presence of sustained FoxO transcriptional activity.^6^ Our results support the finding that reduced mTORC1 signalling in Raptor k.o. mice corresponds to an increased induction of autophagy, as evidenced by a strong reduction in ULK1 phosphorylation and lipidation of LC3. However, this increased induction of autophagy, over time, leads to a block in autophagic flux. This block in both bulk autophagy and mitophagy is possibly due to a significant reduction in FoxO‐dependent gene transcription. Indeed, the hyperactivation of Akt after long‐term deletion is accompanied by a very strong reduction of FoxO‐dependent autophagy‐related genes,^5^ potentially creating a problem in maintaining autophagic flux. Another possible role for mTORC1 signalling in maintaining autophagic flux over time is the fact that during prolonged high levels of autophagy, there is a need to regenerate lysosomes. The process responsible for this is called autophagic‐lysosome reformation and requires mTORC1 re‐activation.^35^ Furthermore, the block in autophagy is critical in the mitochondrial dysfunction, which we already observe at early timepoints after Raptor deletion. Reactivation of autophagy by the Tat‐beclin1 peptide is indeed sufficient to prevent mitochondrial depolarization, likely due to restoration of mitophagic flux.^36^

Taken together, these results suggest that mTORC1 inhibition is important for the induction of autophagy; however, long‐term mTORC1 inhibition leads to a block in autophagic flux.

### Muscle mTORC1 signalling is required for neuromuscular junction maintenance

One of the most surprising findings in this study was the observation that loss of muscle mTORC1 signalling during homeostasis was sufficient to induce fibre denervation. This study adds mechanistic insights to some recently published papers in which genetic alterations in muscle fibres is sufficient to impact the NMJ, without directly affecting the motor neuron.^25^, ^26^ Indeed, these two previous studies showed that inhibiting muscle autophagy or inducing mitochondrial dysfunction in muscle fibres is sufficient to lead to the appearance of denervated fibres. Here, we show that mTORC1 is potentially upstream of these processes, as we find a block in autophagic flux leading to mitochondrial dysfunction and a subsequent appearance of markers of denervation. Interestingly, the loss of spinster, a key protein in autophagic flux and autophagic‐lysosome reformation, leads to significant alterations in the NMJ in drosophila muscle,^37^ linking a proper autophagic flux to NMJ maintenance.

In addition to reducing autophagy, also the inhibition of protein synthesis in muscle fibres is sufficient to affect NMJ stability and the presynaptic component.^38^ Considering the very important reduction in protein synthesis when completely blocking mTORC1 signalling in muscle fibres, it is likely that some of the loss of NMJ stability is due to synthesis of critical muscle proteins for the NMJ. In drosophila, it has been shown that reduction in TOR signalling in muscle fibres is sufficient to compromise synaptic plasticity through retrograde signalling.^39^ Interestingly, this TOR/4E‐BP1‐dependent mechanism acts also during short time periods, like starvation.^40^ The idea that retrograde signalling affects the stability of the motor neuron is in line with the fact that we observe significant alterations of the motor neuron by electron microscope. Multiple factors have been proposed to be secreted by muscle fibres and are important in NMJ maintenance, particularly during stress conditions. For example, FGFBP1 is secreted from muscle fibres during denervation and is particularly important for NMJ maintenance during aging and ALS.^41^, ^42^ Interestingly, approaches targeting neuromuscular diseases like ALS or SBMA with mTORC1 inhibitors, like rapamycin, to activate autophagy in motor neurons, have proven detrimental for disease progression.^11^, ^12^ It is possible that the unexpected negative effect of rapamycin treatment in these pathologies is due to its negative effect on NMJ maintenance by the muscle fibres. In line with this is the fact that muscle‐specific overexpression of IGF1, and therefore mTORC1, is sufficient to significantly improve the phenotype in both pathologies.^13^, ^14^

## Conclusions

Here, we use various genetic and pharmacological loss‐of‐function approaches to show that mTORC1 signalling in adult skeletal muscle plays a critical role in muscle autophagy, force production, and maintenance of the neuromuscular junction. Its role in the NMJ is particularly intriguing, as numerous pathologies affecting the NMJ stability are significantly improved by exercise,^43^ which is known to be a potent activator of mTORC1 signalling in muscle fibres. These findings can contribute to better design future therapeutic interventions, whether it is pharmacological or through exercise, aiming at maintaining mTORC1 activation levels in muscle fibres.

## Author contributions

B. B. conceived the project and wrote the manuscript. M. B., A. G., M. P., L. N., C. T., H. N., V. R., A. M., and S. B. performed experiments and analysed data. M. S. and M. K. contributed intellectually and provided technical advice. All authors discussed the results and commented on the manuscript.

## Conflict of interest

The authors declare that they have no conflict of interest.

## Supporting information


**Figure S1.** **A)** Western blot for 4E‐BP1 and relative quantification. Phosphorylation of 4E‐BP1 on Ser65 and Thr37/46 is reduced upon Raptor deletion **B)** Western blot for IRS‐1 and relative quantification. Increased IRS‐1 protein levels in Raptor k.o. mice 1 month after Raptor deletion **C)** No colocalization between mTOR and Lamp2 (n = 4‐6 muscles/group). Data are shown as mean ± SEM. Statistical analysis was performed using two‐tailed Student t‐test. Statistical significance: *P < 0.05, **P < 0.01
**Figure S2**. Efficient reduction of mTOR transcript (A) and protein level in the inducible mTOR k.o. mice (n = 4‐5 muscles/group). No effect on muscle weight two months after mTOR deletion. Data are shown as mean ± SEM. Statistical analysis was performed using two‐tailed Student t‐test. Statistical significance: *P < 0.05, **P < 0.01, ***P < 0.001
**Figure S3.** No change in epididymal fat or heart weight between wt and Raptor k.o. longterm mice (n = 2‐4 for epididymal fat; n = 6‐8 for heart)
**Figure S4. A)** Volcano plot of the differences in the proteome 1 month after Raptor deletion. Mitochondrial proteins are indicated in red **B)** No reduction in mitochondrial number as evidenced by mitoprofile and blots for TOM20 and SDH (n = 4‐5 muscles/group) **C)** Treadmill performance of Raptor k.o. mice is significantly impaired (n = 4 mice/group). Data are shown as mean ± SEM. Statistical analysis was performed using two‐tailed Student t‐test. Statistical significance: *P < 0.05
**Figure S5**. Expression levels of genes involved in the autophagy‐lysosome system and the ubiquitin‐proteasome system in wildtype and long‐term Raptor k.o. muscles (n = 6/group). Data are shown as mean ± SEM. Statistical analysis was performed using two‐tailed Student t‐test. Statistical significance: *P < 0.05, **P < 0.01
**Figure S6**. Representative image of NCAM‐positive fibers (red) in Raptor k.o. longterm mice
