# Supplementary material for: Skeletal muscle mTORC1 regulates neuromuscular junction stability
Source: J Cachexia Sarcopenia Muscle. 2019 Oct 25;11(1):208–25. doi: 10.1002/jcsm.12496 (PMC7015238; doi:10.1002/jcsm.12496)
Supplement: Supplementary file 1 — Figure S1. A) Western blot for 4E‐BP1 and relative quantification. Phosphorylation of 4E‐BP1 on Ser65 and Thr37/46 is reduced upon Raptor deletion B) Western blot for IRS‐1 and relative quantification. Increased IRS‐1 protein levels in Raptor k.o. mice 1 month after Raptor deletion C) No colocalization between mTOR and Lamp2 (n = 4‐6 muscles/group). Data are shown as mean ± SEM. Statistical analysis was performed using two‐tailed Student t‐test. Statistical significance: *P < 0.05, **P < 0.01 Figure S2. Efficient reduction of mTOR transcript (A) and protein level in the inducible mTOR k.o. mice (n = 4‐5 muscles/group). No effect on muscle weight two months after mTOR deletion. Data are shown as mean ± SEM. Statistical analysis was performed using two‐tailed Student t‐test. Statistical significance: *P < 0.05, **P < 0.01, ***P < 0.001 Figure S3. No change in epididymal fat or heart weight between wt and Raptor k.o. longterm mice (n = 2‐4 for epididymal fat; n = 6‐8 for heart) Figure S4. A) Volcano plot of the differences in the proteome 1 month after Raptor deletion. Mitochondrial proteins are indicated in red B) No reduction in mitochondrial number as evidenced by mitoprofile and blots for TOM20 and SDH (n = 4‐5 muscles/group) C) Treadmill performance of Raptor k.o. mice is significantly impaired (n = 4 mice/group). Data are shown as mean ± SEM. Statistical analysis was performed using two‐tailed Student t‐test. Statistical significance: *P < 0.05 Figure S5. Expression levels of genes involved in the autophagy‐lysosome system and the ubiquitin‐proteasome system in wildtype and long‐term Raptor k.o. muscles (n = 6/group). Data are shown as mean ± SEM. Statistical analysis was performed using two‐tailed Student t‐test. Statistical significance: *P < 0.05, **P < 0.01 Figure S6. Representative image of NCAM‐positive fibers (red) in Raptor k.o. longterm mice [file JCSM-11-208-s001.pdf]

Figure S1

A

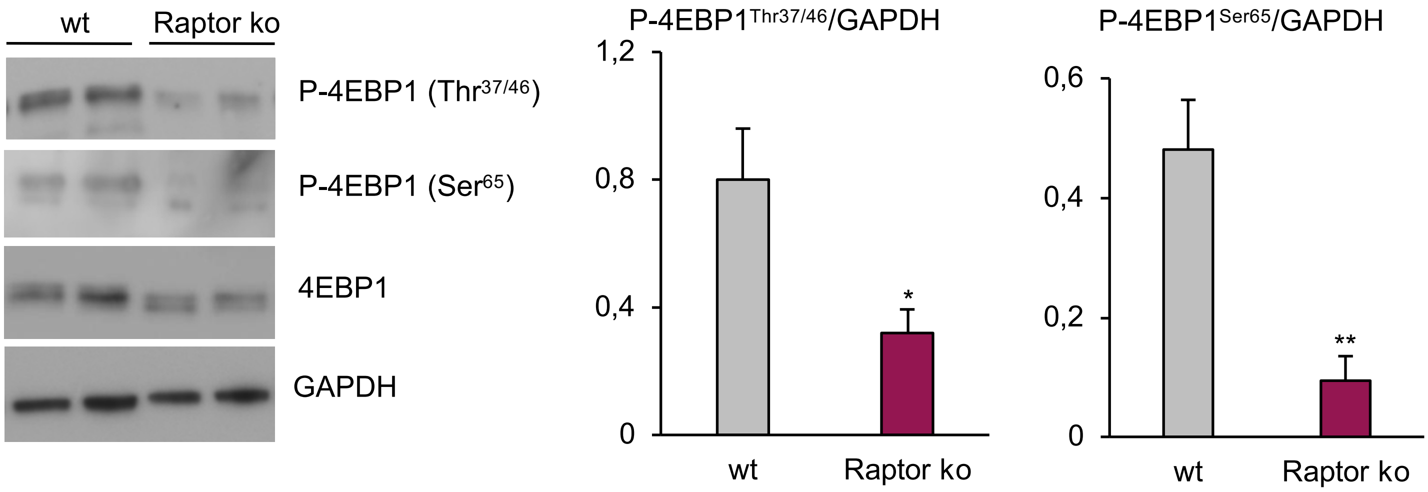

B

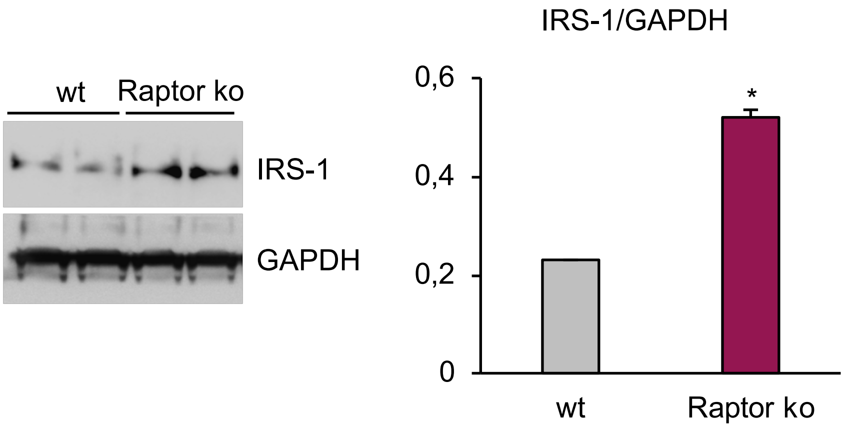

C

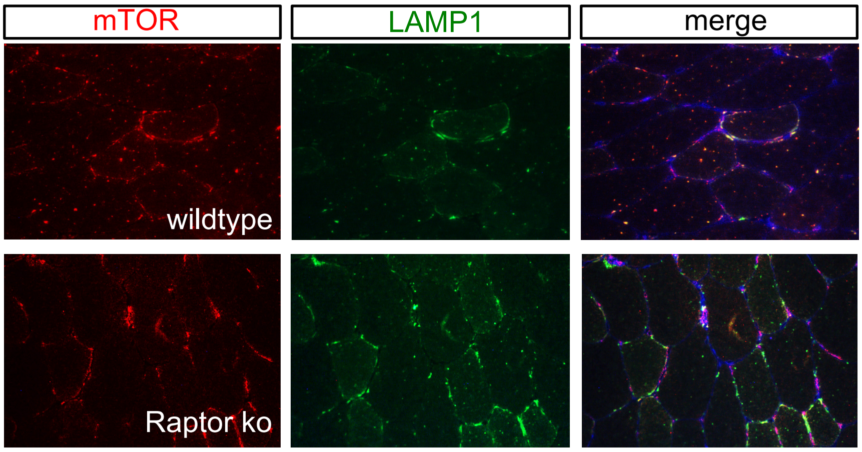

Figure S2

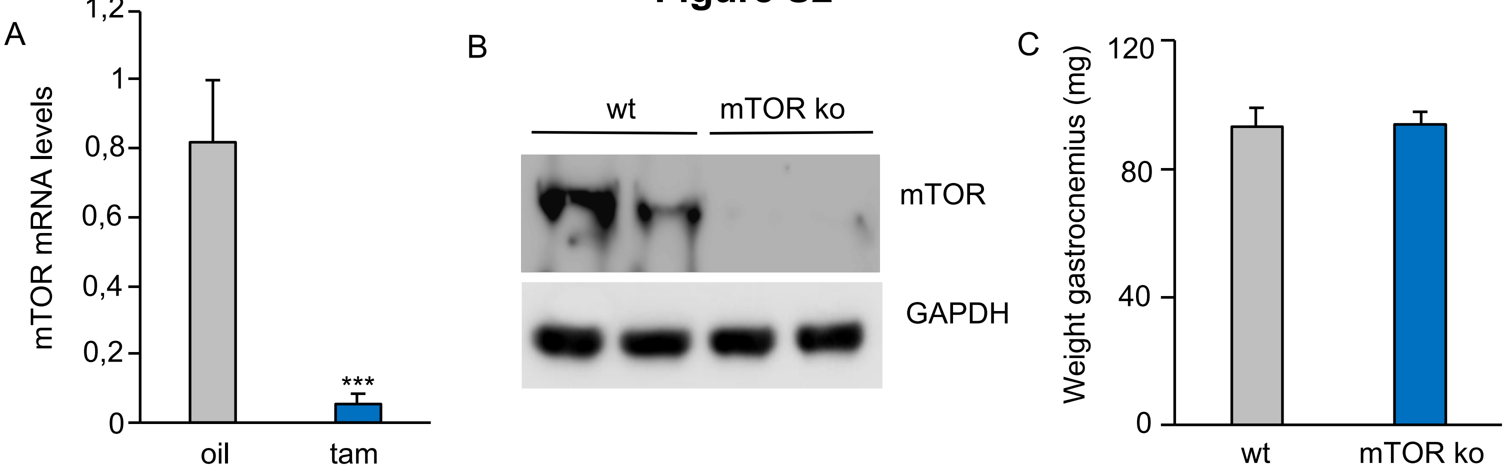
A
mTOR mRNA levels
1,2
1
0,8
0,6
0,4
0,2
0
***
oil
tam
B
wt
mTOR ko
mTOR
GAPDH
C
Weight gastrocnemius (mg)
120
80
40
0
wt
mTOR ko

Figure S3

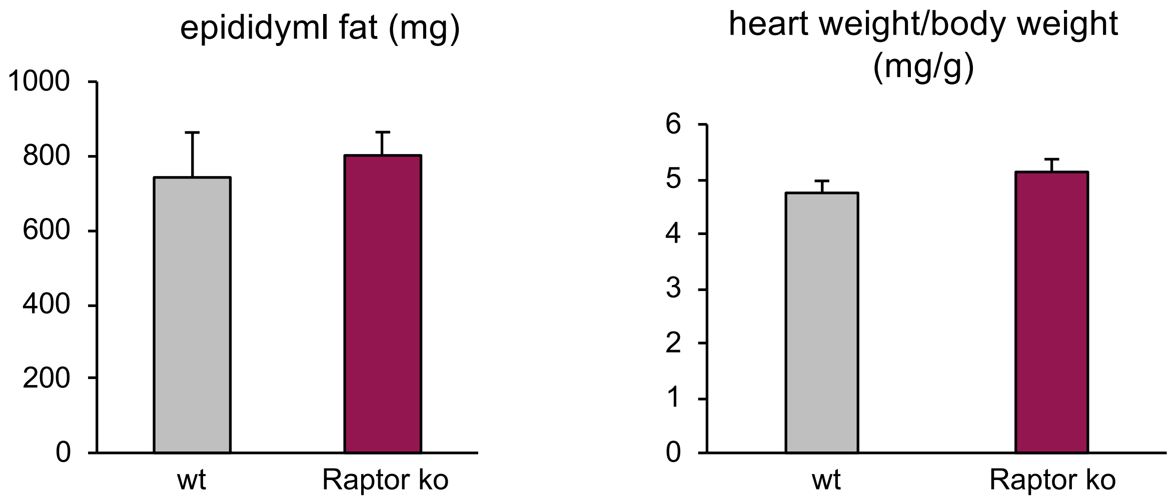
epididyml fat (mg)
1000
800
600
400
200
0
wt
Raptor ko
heart weight/body weight
(mg/g)
6
5
4
3
2
1
0
wt
Raptor ko

Figure S4

A

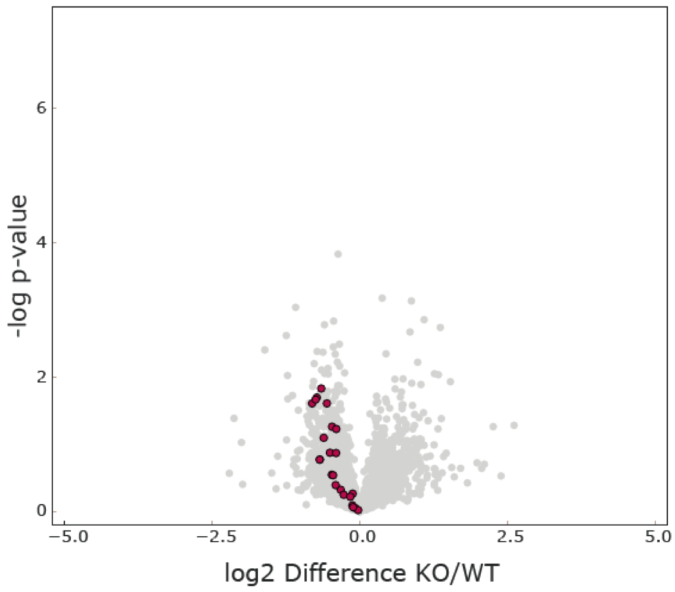

B

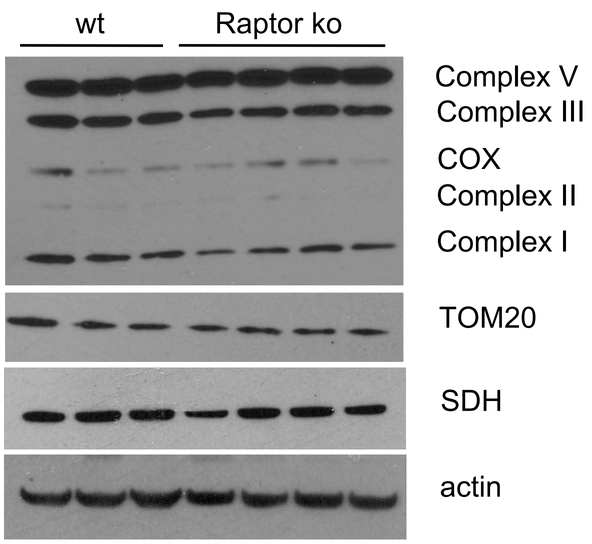

C

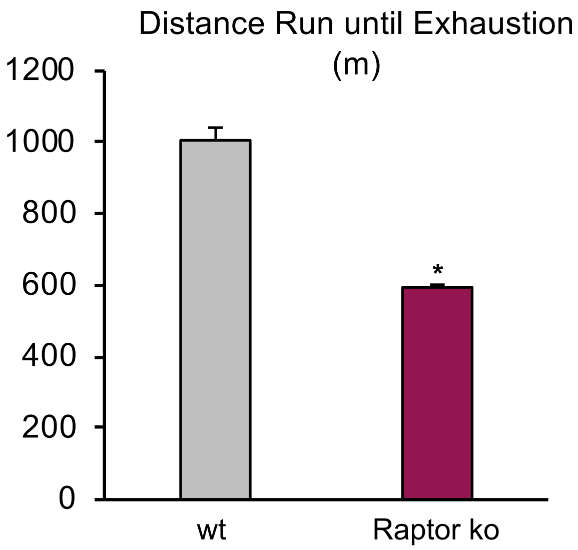

Figure S5

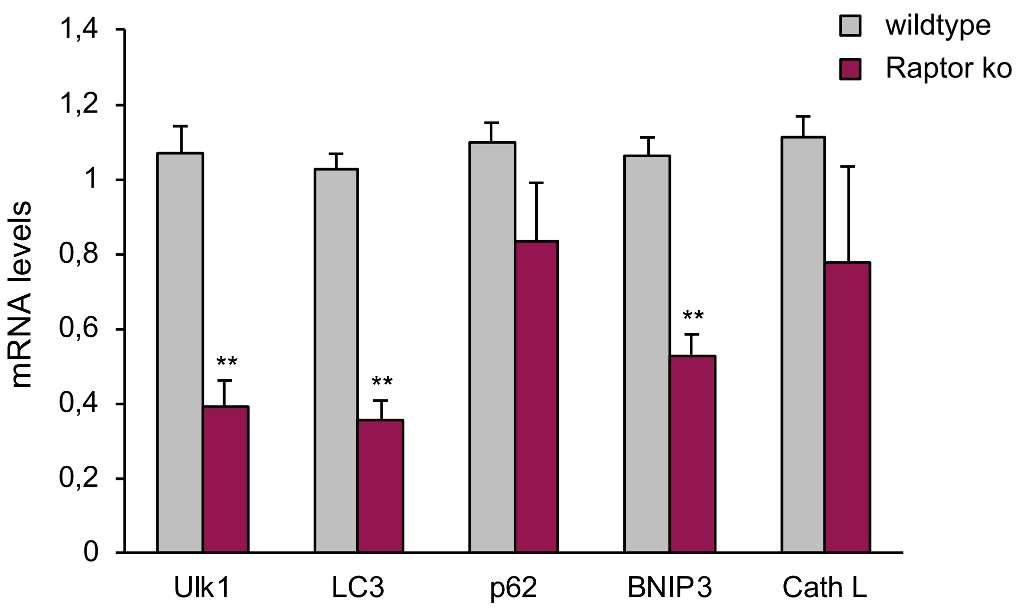

wildtype
Raptor ko
mRNA levels
1,4
1,2
1
0,8
0,6
0,4
0,2
0
**
**
**
Ulk1
LC3
p62
BNIP3
Cath L

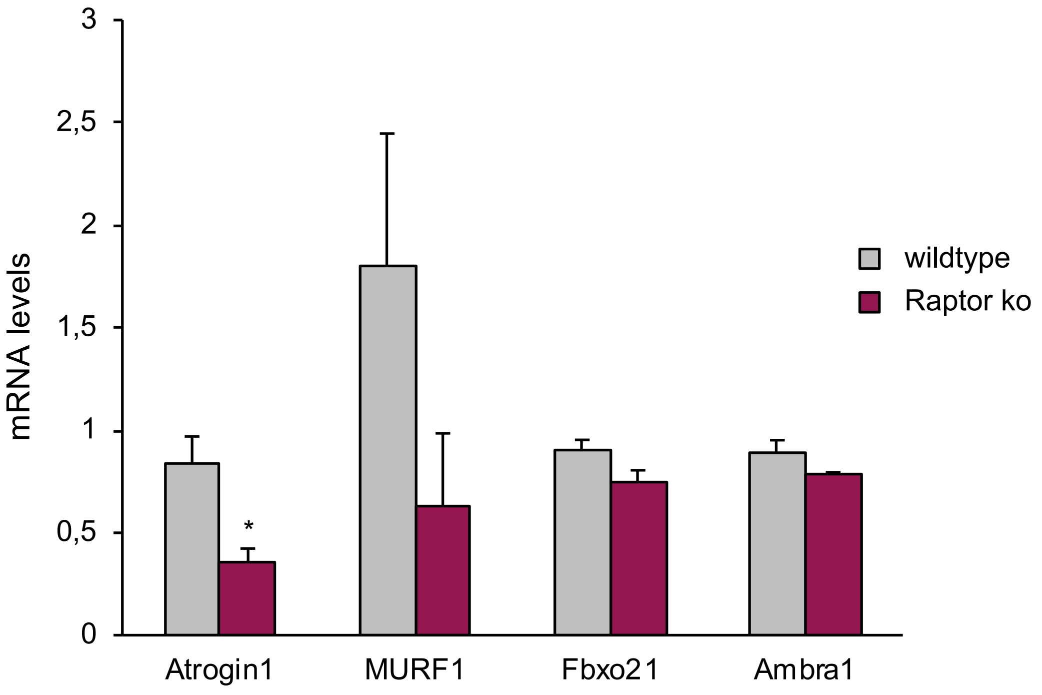

wildtype
Raptor ko
mRNA levels
3
2,5
2
1,5
1
0,5
0
*
Atrogin1
MURF1
Fbxo21
Ambra1

Figure S6

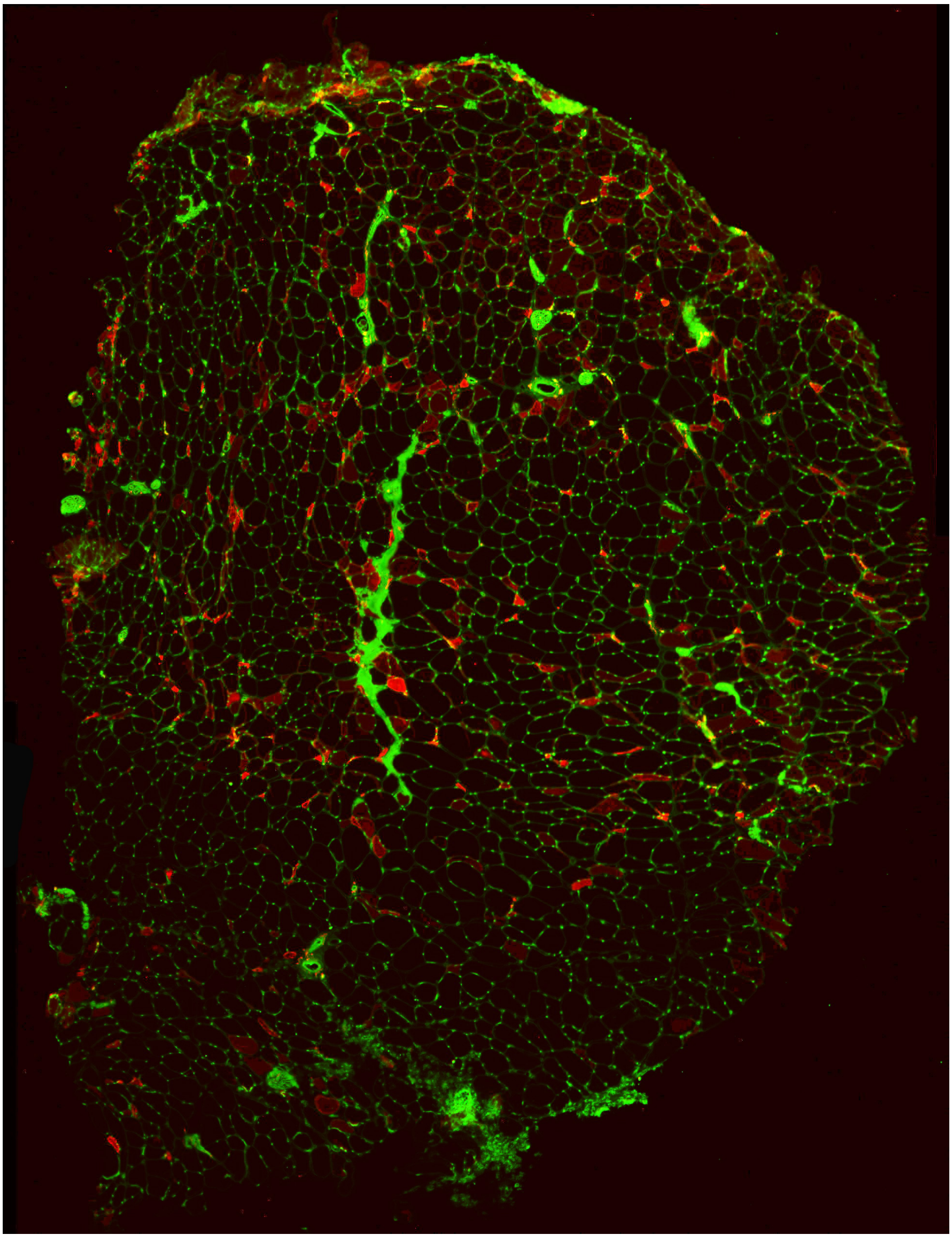

Supporting informations, figure legends

Figure S1. A) *Western blot for 4E-BP1 and relative quantification. Phosphorylation of 4E-BP1 on Ser^{65} and Thr$^{37/46}$ is reduced upon Raptor deletion* ***B)*** *Western blot for IRS-1 and relative quantification. Increased IRS-1 protein levels in Raptor k.o. mice 1 month after Raptor deletion* ***C)*** *No colocalization between mTOR and Lamp2 (n=4-6 muscles/group). Data are shown as mean ± SEM. Statistical analysis was performed using two-tailed Student t-test. Statistical significance: *P<0.05, **P<0.01*

Figure S2*. Efficient reduction of mTOR transcript (A) and protein level in the inducible mTOR k.o. mice (n=4-5 muscles/group). No effect on muscle weight two months after mTOR deletion. Data are shown as mean ± SEM. Statistical analysis was performed using two-tailed Student t-test. Statistical significance: *P<0.05, **P<0.01, ***P<0.001*

Figure S3. *No change in epididymal fat or heart weight between wt and Raptor k.o. longterm mice (n=2-4 for epididymal fat; n=6-8 for heart)*

Figure S4. A) *Volcano plot of the differences in the proteome 1 month after Raptor deletion. Mitochondrial proteins are indicated in red* ***B)*** *No reduction in mitochondrial number as evidenced by mitoprofile and blots for TOM20 and SDH (n=4-5 muscles/group)* ***C)*** *treadmill performance of Raptor k.o. mice is significantly impaired (n=4 mice/group). Data are shown as mean ± SEM. Statistical analysis was performed using two-tailed Student t-test. Statistical significance: *P<0.05*

Figure S5*. Expression levels of genes involved in the autophagy-lysosome system and the ubiquitin-proteasome system in wildtype and long-term Raptor k.o. muscles (n=6/group). Data are shown as mean ± SEM. Statistical analysis was performed using two-tailed Student t-test. Statistical significance: *P<0.05, **P<0.01*

Figure S6*. Representative image of NCAM-positive fibers (red) in Raptor k.o. longterm mice*
